# Effects and safety of the combination of platelet-rich plasma (PRP) and hyaluronic acid (HA) in the treatment of knee osteoarthritis: a systematic review and meta-analysis

**DOI:** 10.1186/s12891-020-03262-w

**Published:** 2020-04-11

**Authors:** Jinlong Zhao, Hetao Huang, Guihong Liang, Ling-feng Zeng, Weiyi Yang, Jun Liu

**Affiliations:** 1 The Second School of clinical medical Sciences, Guangzhou University of Chinese Medicine, Guagnzhou, 510405 China; 2 The Second Affiliated Hospital, Guangzhou University of Chinese Medicine (Guangdong Province Hospital of Traditional Chinese Medicine), Guangzhou, 510120 China; 3 Guangdong Academy of Traditional Chinese Medicine, Research Team on Bone and Joint Degeneration and Injury, Guangzhou, 510120 China

**Keywords:** Platelet-rich plasma, Hyaluronic acid, Knee osteoarthritis, Meta-analysis

## Abstract

**Background:**

Studies have shown that the combined application of hyaluronic acid (HA) and platelet-rich plasma (PRP) can repair degenerated cartilage and delay the progression of knee osteoarthritis (KOA). The purpose of this study was to explore the efficacy and safety of the intra-articular injection of PRP combined with HA compared with the intra-articular injection of PRP or HA alone in the treatment of KOA.

**Methods:**

The PubMed, Cochrane Library, EMBASE and China National Knowledge Infrastructure (CNKI) databases were searched from inception to December 2019. Randomized controlled trials and cohort studies of PRP combined with HA for KOA were included. Two orthopaedic surgeons conducted the literature retrieval and extracted the data. Outcome indicators included the Western Ontario and McMaster Universities Arthritis Index (WOMAC), the Lequesne Index, the visual analogue scale (VAS) for pain, and adverse events (AEs). Review Manager 5.3 was used to calculate the relative risk (RR) or standardized mean difference (SMD) of the pooled data. STATA 14.0 was used for quantitative publication bias evaluation.

**Results:**

Seven studies (5 randomized controlled trials, 2 cohort studies) with a total of 941 patients were included. In the VAS comparison after 6 months of follow-up, PRP combined with HA was more likely to reduce knee pain than PRP alone (SMD: − 0.31; 95% confidence interval (CI): − 0.55 to − 0.06; *P* = 0.01 < 0.05). PRP combined with HA for KOA achieved better improvements in the WOMAC Function Score (SMD: -0.32; 95% CI: − 0.54 to − 0.10; *P* < 0.05) and WOMAC Total Score (SMD: -0.42; 95% CI: − 0.67 to − 0.17; *P* < 0.05) at the 12-month follow-up than did the application of PRP alone. In a comparison of Lequesne Index scores at the 6-month follow-up, PRP combined with HA improved knee pain scores more than PRP alone (SMD: -0.42; 95% CI: − 0.67 to − 0.17; *P* < 0.05). In terms of AEs, PRP combined with HA was not significantly different from PRP or HA alone (*P* > 0.05).

**Conclusions:**

Compared with intra-articular injection of PRP alone, that of PRP combined with HA can improve the WOMAC Function Scores, WOMAC Total Score, 6-month follow-up VAS ratings, and Lequesne Index scores. However, in terms of the incidence of AEs, PRP combined with HA is not significantly different from PRP or HA alone.

## Background

Knee osteoarthritis (KOA) is a common knee degenerative disease characterized by cartilage degeneration, cartilage exfoliation, and subchondral bone hyperplasia, leading to knee pain, joint instability and functional limitations [1]. KOA severely affects patients’ quality of life and is a major public health issue [2]. An epidemiological survey published in Proceedings of the National Academy of Sciences (PNAS) showed that the incidence of KOA in the U.S. population has doubled since the mid-twentieth century [3]. KOA has become a high-incidence human disease and has caused a great negative impact on people’s lives and work.

The Osteoarthritis Society International (OARSI) recommends conservative treatment rather than surgery as the first-line management solution for KOA, which emphasizes the importance of conservative treatment in the treatment of KOA [4]. The American College of Rheumatology (ACR) has proposed a classification in which conservative treatment includes drug treatment and non-drug treatment [5]. Non-drug treatment includes general exercise and muscle exercise, but non-drug methods often depend heavily on patient compliance and are difficult to control [5]. The main drug therapies include analgesics, non-steroidal anti-inflammatory drugs and corticosteroid injections [6]. Although the above drug therapies are effective to a certain degree, there are also major side effects [6, 7]. In recent years, there have been an increasing number of studies on the application of intra-articular injection of platelet-rich plasma (PRP) or hyaluronic acid (HA) in the treatment of KOA. Many systematic reviews suggest that intra-articular injection of PRP, compared to HA, can alleviate pain symptoms and improve knee function in patients with KOA [6, 8, 9]. However, a double-blind randomized controlled trial with a 5-year follow-up showed that the combination of HA and PRP improved pain and function in patients with a history of chronic symptomatic knee degenerative changes and osteoarthritis [10]. An RCT showed that PRP is an effective treatment for mild to moderate KOA and that the combined use of HA and PRP is better than the use of HA (1 year) and PRP (3 months) alone [11]. The RCT also revealed that PRP does not provide better overall clinical improvement than HA in terms of symptom-function improvement at different follow-up points or in terms of duration of effect [10]. In recent years, an increasing number of studies have focused on the rationality of PRP combined with HA for KOA, and their mechanisms have been discussed in depth. Experimental studies comparing the migration capabilities of tendon cells and synovial fibroblasts in pure PRP solution and PRP plus HA solution have shown that mixing PRP with HA can significantly improve cell mobility [12]. Marmotti found that the addition of HA to PRP can effectively promote the proliferation of chondrocytes and improve the ability of cartilage repair [13]. Studies have shown that the combination of PRP and HA may benefit from its different biological mechanisms and facilitate the activity of signal molecules such as inflammatory molecules, catabolic enzymes, cytokines and growth factors, thereby playing a positive role in the treatment of KOA [11, 14]**.**

In recent years, clinical workers have begun treating KOA with intra-articular injections of HA combined with PRP to take advantage of their synergistic therapeutic effects. The purpose of this study was to explore the efficacy and safety of intra-articular injection of PRP combined with HA compared with PRP or HA alone, providing an evidence-based strategy for the treatment of KOA.

## Methods

This meta-analysis was performed strictly in accordance with the relevant requirements of the Preferred Reporting Items for Systematic Reviews and Meta-Analyses (PRISMA) Statement.

### Inclusion and exclusion criteria

Inclusion criteria. (1) Type of study: Published randomized controlled trial (RCT) or cohort study. (2) Research subjects: Individuals with a clear diagnosis of KOA, regardless of age, gender or nationality. (3) Intervention: Administration of intra-articular injection of PRP combined with Ha to the test group and intra-articular injection of PRP or HA to the control group. Two- or three-arm studies were eligible. (4) At least one of the following outcome indicators: Western Ontario and McMaster Universities Arthritis Index (WOMAC), Lequesne Index, visual analogue scale (VAS), and adverse events (AEs). (5) No application of language exclusions.

Exclusion criteria. (1) Reviews, meeting abstracts, case reports. (2) Subjects with both KOA and hip osteoarthritis. (3) Studies in which the intervention did not include intra-articular injection of PRP combined with HA. (4) Duplicate publications or studies with similar data. (5) Incomplete, unclear, or obviously erroneous data that could not be resolved by contacting the authors.

### Literature retrieval strategy

The PubMed, Cochrane Library, EMBASE and China National Knowledge Infrastructure (CNKI) databases were searched, and RCTs and cohort studies that met the inclusion criteria were included. The retrieval period was from the establishment of each database to December 2019. Two researchers also performed cross-referencing to reduce retrieval errors. The search terms included “platelet-rich plasma”, “PRP”, “hyaluronic acid”, “HA”, “knee osteoarthritis” and “KOA”. See Supplement 1 for the database retrieval strategies.

### Literature screening and data extraction

Two orthopaedic surgeons conducted the literature retrieval; the preliminary and secondary screenings of the literature were performed strictly in accordance with the pre-established inclusion and exclusion criteria. The two researchers extracted the data independently, and a third researcher compared their outputs. In the event of an error or difference, the third researcher and corresponding author assisted in the judgement.

The main data extracted in this study included first author, publication year, sample size, intervention measures, ethical approval, gender, age, BMI, follow-up periods, radiographic classification, relevant items for literature quality evaluation and relevant outcome indicators of clinical efficacy and safety.

### Risk of bias assessment of the included studies

Regarding RCTs, the Cochrane risk of bias tool was used for quality evaluation [15]. The tool includes evaluation in seven areas: random sequence generation, allocation blinding, blinding of participants, blinding of outcome measures, incomplete outcome data, selective reporting, and other biases. The risk of bias in each area was judged to be low, high or unclear [16]. For cohort studies, the Newcastle-Ottawa Scale (NOS) was used for quality assessment. This scale includes three aspects: (1) selection of study groups; (2) ascertainment of exposure and outcome; and (3) group comparability. Studies with scores greater than or equal to 7 were considered to have a low risk of bias, scores of 4 to 6 indicated a moderate risk of bias, and scores less than 4 indicated a high risk of bias.

### Statistical analysis

The relative risk (RR) was used to evaluate the effects of binary variables, the standardized mean difference (SMD) was used to evaluate the effects of continuous variables, and 95% confidence intervals (CIs) of the RR and SMD were calculated. Review Manager 5.3.5 software (Cochrane Collaboration, Oxford, UK) was used to calculate the efficacy and safety indicators and their 95% CIs. In addition, for homogeneous data sets, *P* > 0.1 and I^2^ < 50% were used as the test standards. When the above two statistical conditions were met, a fixed-effects model was used for the meta-analysis because the pooled effect sizes were relatively homogenous. If one of the above standards did not conform, the homogeneity of the pooled effect size was not ideal, and a random-effects model was applied.

To quantitatively assess whether there are publication biases in different outcome indicators, this study used Stata 14.0 (STATA Corporation, Lakeway, Texas, USA) software to perform Egger’s and Begg’s linear regression tests on the outcome indicators included in the combined analysis of three or more studies.

## Results

### Literature screening process and results

**Fig. 1:**
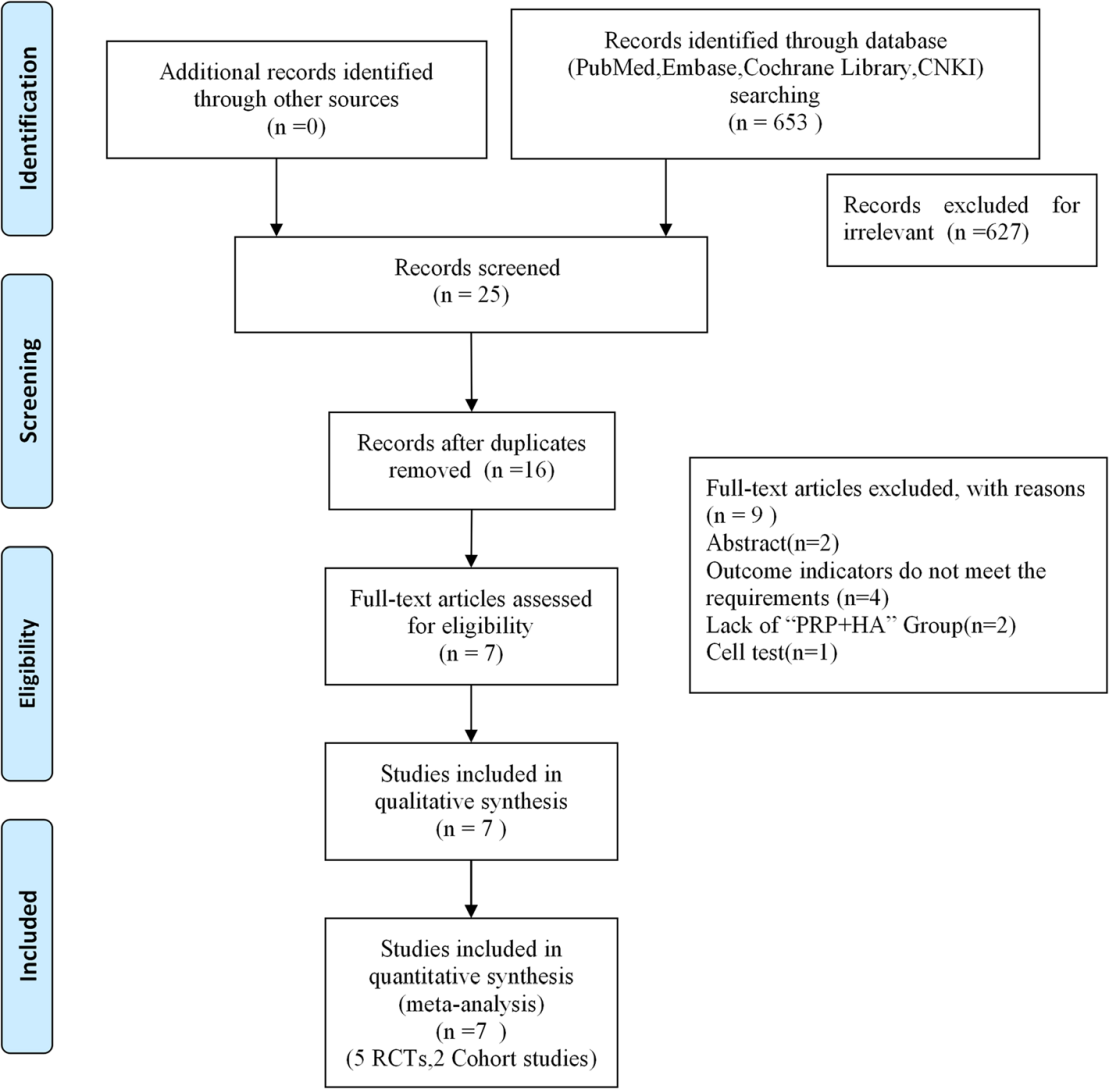
Flow diagram of study selection

A total of 653 related studies were obtained in the preliminary inspection, including *n* = 170 from PubMed, *n* = 218 from Embase, *n* = 128 from the Cochrane Library, *n* = 37 from CNKI, and *n* = 0 from other manual searches. After reading the titles and abstracts and excluding irrelevant documents, a total of 27 articles remained. After excluding duplicate studies, following the inclusion criteria and exclusion criteria, this study eventually included 5 RCTs and 2 cohort studies; all 7 studies clearly stated that they had received ethical approval. The included literature included two three-arm trials, and the rest were two-arm trials, for a total of 941 patients. The literature screening process and results are shown in Fig. 1. Analysing the basic characteristics of the included cases, it was found that the age of the included cases was mainly concentrated in the 40–60 year range, the Kellgren and Lawrence grading scale was I to IV, and the follow-up time was 6–12 months. The basic information of the included literature is shown in Table 1. Baseline materials such as age, BMI, and sample size of the patients included in the 7 studies were comparable, all with *p > 0.05*. The preparation of PRP combined with HA and the dosage, frequency, and duration of treatment of the 7 studies included were systematically summarized. Of the 7 studies included in this study, the PRP used by patients was derived from the patient’s own whole blood. PRP is a blood product containing high concentrations of platelets, white blood cells and a large number of growth factors produced by whole blood centrifugation. The amount of PRP per serving was approximately 2–8 ml. The frequency of injection of PRP and HA therapy was once a week for 3–9 weeks, which has certain theoretical significance for clinical application. There were some differences in PRP concentrations in the literature included in this study, which may have had an impact on the efficacy of treating KOA. The results are shown in Supplement 2.

**Table 1:** Characteristics of the included studies

Author,year	Study	Ethical approval	Sample Size			Gender (M/F)			Age, years			BMI(kg/m2)			Radiographic Classification (K-L)	Clinical Outcomes	Follow-up Periods
			PRP + HA	PRP	HA	PRP + HA	PRP	HA	PRP + HA	PRP	HA	PRP + HA	PRP	HA			
Michele Abate 2015 [17]	Co,R	Yes	40	40	–	31/9	21/ 19	–	56.7± 11.2	60.9 ± 9	–	23.7 ± 2	24.1± 1.6	–	II-III	VAS,AE	6 ms
Wenxing Yu 2018 [18]	RCT	Yes	96	104	88	50/46	50/ 54	48/40	46.5± 7.5	46.2 ± 8.6	51.5 ± 9.3	–	–	–	I,II,III,IV	WOMAC, AE	12 ms
Yanqing Guo 2016 [19]	Co,P	Yes	63	63	–	18/45	12/ 51	–	61.2± 9.6	60.7 ± 10.1	–	24.2 ± 4.2	24.6± 3.9	–	I,II,III	VAS,AE, WOMAC	12 ms
Quanwei Ding 2017 [20]	RCT	Yes	20	27	–	2/18	8/ 19	–	56.75± 9.536	62.11 ± 12.5	–	24.31± 3.764	24.27 ± 3.636	–	I,II,III	WOMAC, VAS, Lequesne scores	6 ms
Xin-liang Zhao 2018 [21]	RCT	Yes	62	62	–	35/27	37/ 25	–	55.73 ± 7.18	56.32 ± 8.13	–	–	–	–	–	VAS	1 month
Yanqing Guo 2018 [22]	RCT	Yes	63	63	–	45/18	51/ 12	–	61 ± 10	61 ± 10	–	24 ± 4	24 ± 4	–	I,II,III	VAS,AE	12 ms
Chenrong Ke 2016 [23]	RCT	Yes	50	50	50	25/25	24/ 26	23/27	57.8 ± 6.9	59.3 ± 7.1	58.8 ± 6.4	–	–	–	I,II,III,IV	Lequesne scores,AE	12 ms

*Co* Cohort study, *P* prospective study, *R* retrospective study, *RCT* randomized control trail, *BMI* body mass index, *HA* hyaluronic acid, *K-L* Kellgren and Lawrence grading scale, *PRP* platelet-rich plasma, *AE* Adverse events, *VAS* visual analog scale, *WOMAC* Western Ontario and McMaster Universities Arthritis Index, *ms* months

### Quality evaluation of the included literature

#### Quality evaluation of the 5 RCTs

**Fig. 2:**
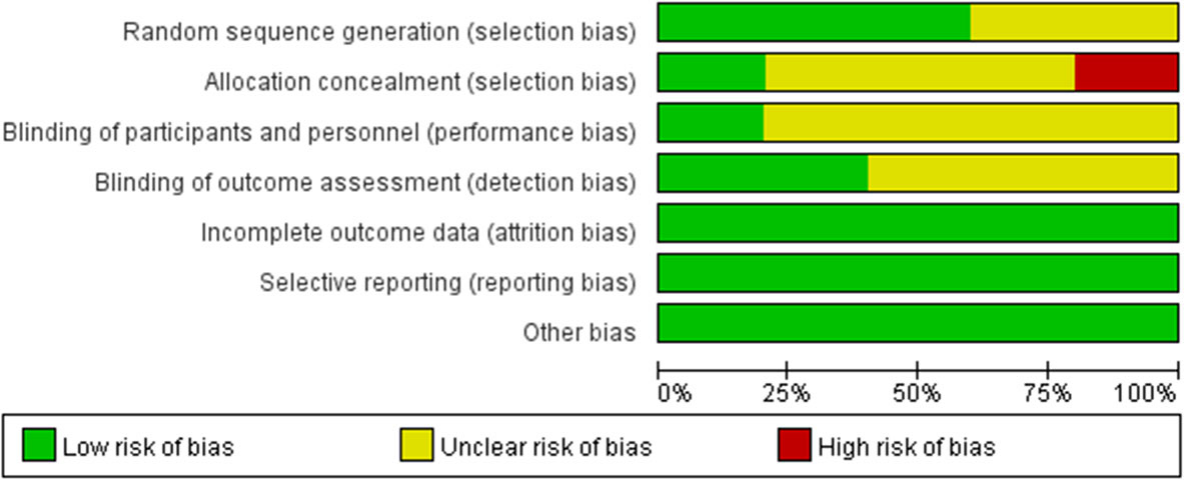
Risk of bias assessment

**Fig. 3:**
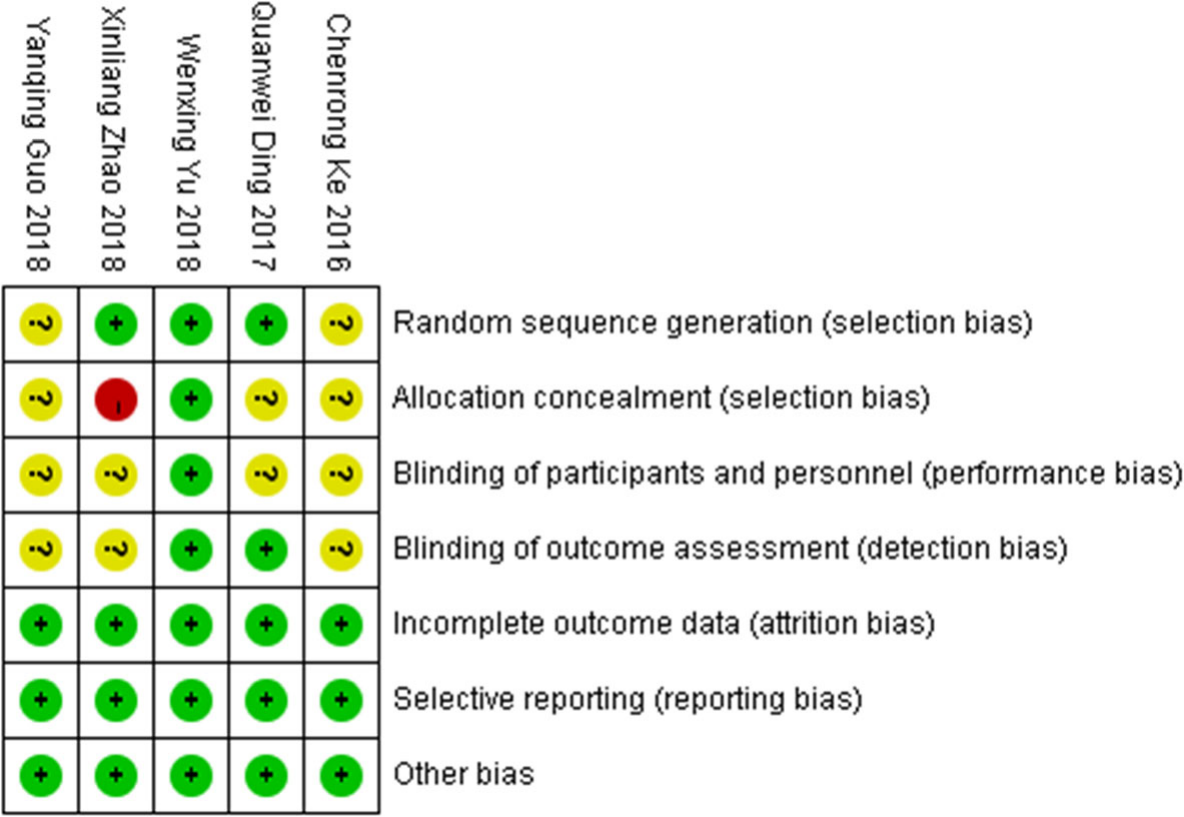
Risk of bias summary

There were 3 studies that explicitly reported the specific method of using random allocation, such as the random number table method, and 2 studies merely mentioned randomness and did not explain the specific method. Three papers did not explain the allocation and concealment, and 1 paper did not perform allocation and concealment. The blinding risk of the participants in the blind method and the outcome index measurement process was mainly unclear risk and low risk, and no high risk was found in the literature. None of the five RCTs had missing data, selective reporting, or other risks (Fig. 2 and Fig. 3).

#### Quality evaluation of the 2 cohort studies

The NOS scores of both cohort studies were 9, and both were low risk (Table 2). In general, the 7 articles included were of good quality, with a standardized research design and good research value.

**Table 2:** Newcastle-Ottawa Scale for Risk of Bias Assessment of Cohort Studies Included in the Meta-Analysis

Study	Selection				Comparability	Outcome			Overall
	Representativeness of Exposed Cohort	Selection of Nonexposed	Ascertainment of Exposure	Outcome Not Present at Start		Assessment of Outcome	Adequate Follow- Up Length	Adequacy of Follow-Up	
Michele Abate et al. 2015 [17]	★	★	★	★	★★	★	★	★	9
Yanqing Guo et.al 2016 [19]	★	★	★	★	★★	★★	★	☆	9

★,score of 1; ★★, score of 2; ☆,score of 0

### Meta-analysis

#### Vas

**Fig. 4:**
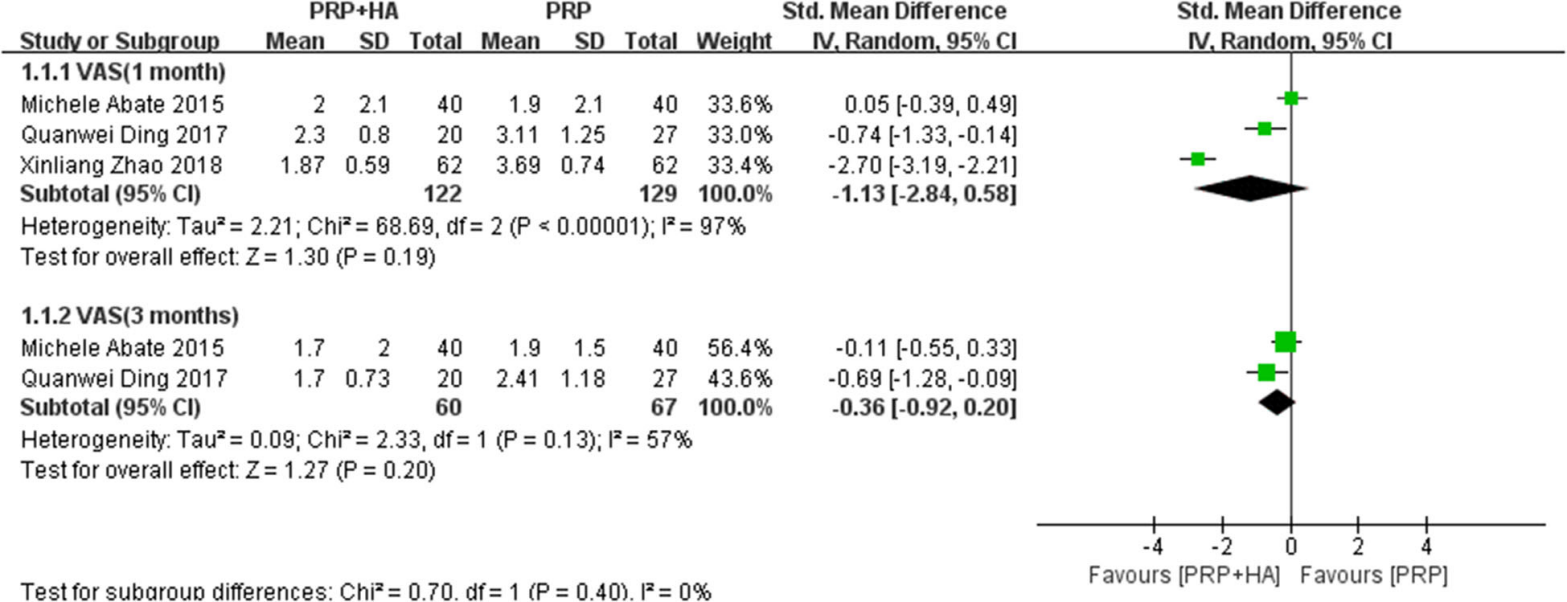
Forest plot and meta-analysis of VAS score(1 and 3 months)

A total of 3 studies reported VAS scores at 1 month after treatment. The heterogeneity test indicated that the homogeneity was not ideal (I^2^ = 97%, *P* < 0.00001). A random-effects model was used for the meta-analysis. The results showed that PRP combined with HA was not significantly different from PRP alone (SMD: -1.13, 95% CI: − 2.84 to 0.13, *P* = 0.19 > 0.05) (Fig. 4).

A total of 2 articles reported VAS scores at 3 months after treatment. The heterogeneity test indicated heterogeneity (I^2^ = 57%, *P* = 0.13), and a random-effects model was used for meta-analysis. The results showed that PRP combined with HA was not significantly different from PRP alone (SMD: -0.36, 95% CI: − 0.92 to 0.20, *P* = 0.20 > 0.05) (Fig. 4).

**Fig. 5:**
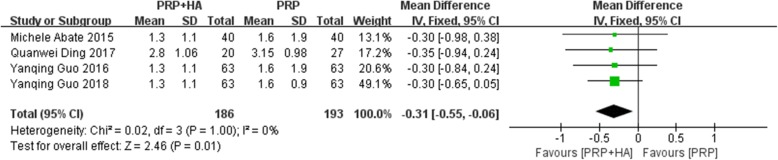
Forest plot and meta-analysis of VAS score(6 months)

A total of 4 studies reported VAS scores at 6 months after treatment. The heterogeneity test suggested a high degree of homogeneity (I^2^ = 0%, *P* = 1.00), and a fixed-effects model was used for meta-analysis. The results showed that the difference between PRP combined with HA compared with PRP alone was statistically significant (SMD: -0.31, 95% CI: − 0.55 to − 0.06, *P* = 0.01 < 0.05) (Fig. 5). The results showed that at 6 months after treatment, the group receiving PRP combined with HA had an average VAS score that was 0.31 points lower than that of the group receiving PRP alone.

#### WOMAC function score

**Fig. 6:**
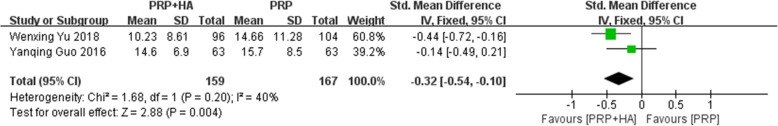
Forest plot and meta-analysis of WOMAC Function Score

A total of 2 studies reported the WOMAC Function Score at 12 months after treatment. The heterogeneity test showed good homogeneity (I^2^ = 40%, *P* = 0.20), and a fixed-effects model was used for the meta-analysis. The results showed that the difference between PRP and HA compared with PRP alone was statistically significant (SMD: -0.32, 95% CI: − 0.54 to − 0.10, *P* = 0.004 < 0.05) (Fig. 6). The results showed that at 12 months after treatment, the WOMAC Function Score of the group receiving PRP combined with HA was 0.32 points lower than that of the group receiving PRP alone.

#### WOMAC Total score

**Fig. 7:**
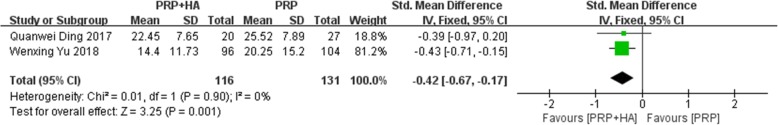
Forest plot and meta-analysis of WOMAC Total Score

A total of 2 studies reported comparisons of the WOMAC Total Score at 12 months after treatment. The heterogeneity test indicated that the homogeneity was good (I^2^ = 0%, *P* = 0.90), and a fixed-effects model was used for meta-analysis. The results showed that the difference between PRP combined with HA compared with PRP alone was statistically significant (SMD: -0.42, 95% CI: − 0.67 to − 0.17, *P* = 0.001 < 0.05) (Fig. 7). The results showed that at 12 months after treatment, the WOMAC Total Score of the group receiving PRP combined with HA was 0.42 points lower than that of the group receiving PRP alone.

#### Lequesne index

**Fig. 8:**
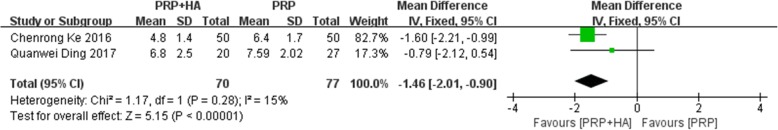
Forest plot and meta-analysis of Lequesne Index scores

A total of 2 studies reported Lequesne Index scores 6 months after treatment. The heterogeneity test indicated that the homogeneity was good (I^2^ = 15%, *P* = 0.28), and a fixed-effects model was used for meta-analysis. The results showed that PRP combined with HA had significant differences compared with PRP alone (SMD: -0.42, 95% CI: − 0.67 to − 0.17, *P* < 0.00001) (Fig. 8). The results showed that at 6 months after treatment, the Lequesne Index of the group receiving PRP combined with HA was reduced by 0.42 points compared with that of the group receiving PRP alone.

#### AEs

**Fig. 9:**
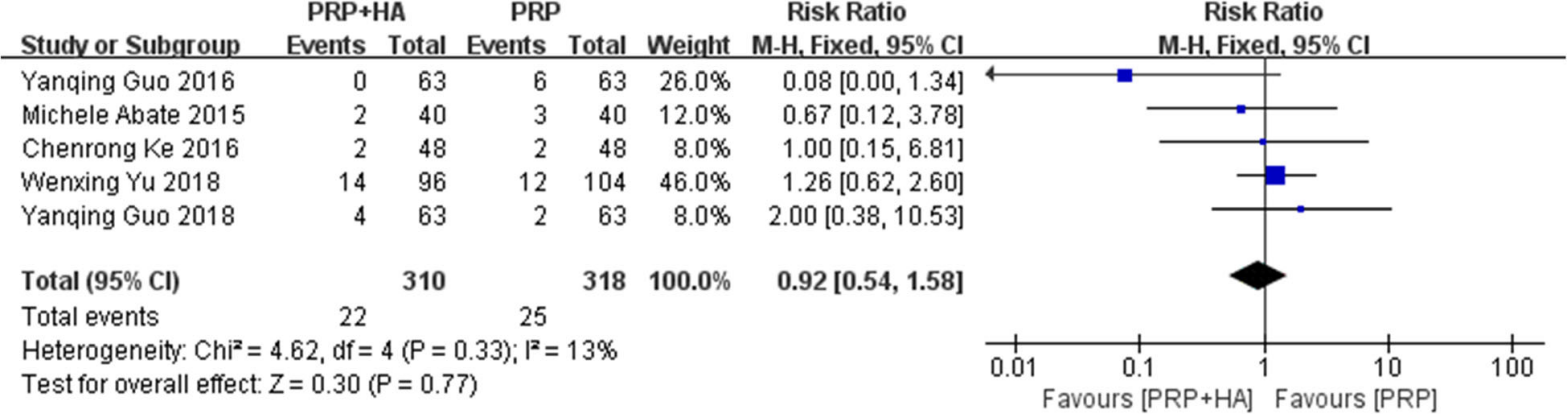
Forest plot and meta-analysis of Adverse events (PRP + HA VS PRP)

A total of 5 studies reported the comparison of AEs of PRP combined with HA and PRP alone on KOA. The heterogeneity test showed that the homogeneity was good (I^2^ = 13%, *P* = 0.33), and a fixed-effects model was used for meta-analysis. The results showed no significant difference between PRP combined with HA compared with PRP alone (RR: 0.92, 95% CI: 0.54 to 1.58, *P* = 0.77) (Fig. 9). The main types of adverse reactions were pain, proteinuria, redness, peripheral oedema, constipation and worsening of pain, without serious adverse reactions.

**Fig. 10:**
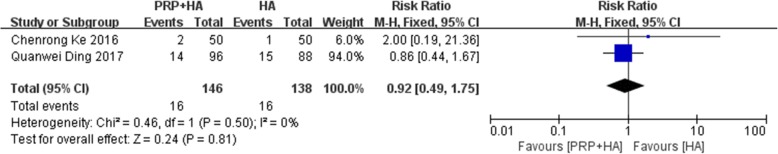
Forest plot and meta-analysis of Adverse events (PRP + HA VS HA)

A total of 2 studies reported the comparison of AEs of PRP combined with HA and the application of HA alone in the treatment of KOA. The heterogeneity test suggested a high degree of homogeneity (I^2^ = 0%, *P* = 0.50), and a fixed-effects model was used for meta-analysis. The results showed that PRP combined with HA had no significant difference compared with the application of HA alone (RR: 0.92, 95% CI: 0.49 to 1.75, *P* = 0.81) (Fig. 10).

### Evaluation of publication bias

To quantitatively analyse whether there are publication biases in the relevant outcome indicators of this study, Egger’s and Begg’s tests were conducted on the outcome indicators combined with 3 or more studies. The results showed that there was no publication bias in the results of VAS after 1 month of treatment (Begg’s test: Pr>|z| = 1.000>0.05; Egger’s test: *P* = 0.857>0.05), VAS after 6 months of treatment (Begg’s test: Pr>|z| = 0.734>0.05; Egger’s test: *P* = 0.619>0.05), and AEs (Begg’s test: Pr>|z| = 0.221>0.05; Egger’s test: *P* = 0.269>0.05). The data analysis process and statistical results of publication bias are shown in Supplementary 3.

## Discussion

KOA is a disease that can cause lower extremity disability, reduce the quality of life of patients, and seriously affect the physical and mental health of middle-aged and elderly people [24]. With the ageing of the population, KOA will gradually become a common and frequently occurring disease, which is a major challenge that health systems in various countries need to meet [25]. The pathogenesis of KOA is still unclear, and there is still no continuous and effective conservative treatment [10]. For patients with KOA who are successfully treated with conservative treatment, surgical treatment is mostly used. However, surgical treatment is mostly used for patients with severe KOA [26]. In addition, although PRP combined with HA for KOA may be complicated and even more expensive, perhaps compared with the cost and risk of surgery, PRP combined with HA may be a better choice. However, a cost-effectiveness study of PRP combined with HA for KOA and PRP or HA alone is still lacking and needs further research. Moreover, surgical treatment has a long recovery period, unavoidable risks of surgery and complications [27]. Therefore, it is of great significance to study new treatments for KOA. In recent years, intra-articular injection of PRP or HA for the treatment of KOA has attracted strong interest from many clinicians, and in-depth research has been conducted [28, 29].

PRP is extracted by centrifugation from autologous blood, and the platelet concentration can be increased nearly 10-fold, which contains approximately 1500 proteins that can release macrophages and growth factors after activation, which is beneficial not only for removing necrotic tissue and reducing the inflammatory response but also for articular cartilage repair and regeneration [30–32]. HA is a high molecular weight polysaccharide that is an important part of synovial fluid and articular cartilage. Injecting HA into the knee joint cavity can physically lubricate the articular surface, reduce wear, and biologically nourish articular cartilage and promote the synthesis of endogenous HA, thereby delaying further joint disease [33–35]. A large number of RCTs and systematic reviews of the intra-articular injection of PRP or HA for KOA have been published [8, 36–38], and most studies have concluded that intra-articular injection of PRP, compared with HA, can relieve knee pain and improve the function of patients with KOA. Moreover, research has shown that the combined application of PRP and HA can repair the degeneration of cartilage and delay the progression of KOA [11, 14, 39]. This synergistic effect mainly changes the role of inflammatory cytokines in the process of chondrocyte degeneration through specific mediators (CD44, TGF-βRII), thereby promoting cartilage regeneration and inhibiting the inflammatory response [40].

In this study, a meta-analysis showed that there was no significant difference between PRP combined with HA and PRP alone for KOA at 1 month or 3 months after treatment. This outcome shows that the effects of the two intervention methods in relieving knee pain are similar at 1 month and 3 months after treatment. However, the VAS score at 6 months after treatment showed that intra-articular injection of PRP combined with HA, compared with PRP alone, could relieve pain in patients with KOA. Intra-articular injection of PRP combined with HA has a unique advantage in the long-term relief of pain in patients with KOA, but the results from longer follow-up periods are still needed for comparison. This meta-analysis found that PRP combined with HA at 6 months after treatment was superior to PRP alone, which may suggest that PRP combined with HA may be a better treatment for patients with long-term knee pain in the future. Previous studies have shown that PRP combined with HA for KOA can reduce WOMAC pain scores more than PRP alone can, which is consistent with the present findings [18]. In terms of the improved WOMAC Function Score and WOMAC Total Score, intra-articular injection of PRP combined with HA, compared with PRP alone, can improve patients’ knee joint function scores and overall WOMAC scores. Studies have reported that compared with PRP alone, HA combined with PRP in KOA treatment significantly improves physical function at 1 and 3 months after treatment [11]. From the analysis of Lequesne Index scores, it was found that PRP combined with HA reduced the Lequesne Index scores more than PRP alone did, which showed that PRP was more effective in relieving knee pain. This systematic review compared and analysed the adverse reactions of PRP combined with HA compared with PRP and HA alone for KOA. The results showed that no significant difference was found in the incidence of adverse reactions, whether PRP, HA, or both were applied, indicating that the safety of the three treatments was not different. The meta-analysis conducted Begg’s and Egger’s tests on VAS after 1 month and 6 months of treatment and AEs. The results suggest that there was no publication bias, indicating that the above results are reliable.

This study is the first systematic evaluation of the efficacy and safety of PRP combined with HA compared with that of PRP or HA alone for KOA. Additionally, this meta-analysis systematically summarizes the preparation process of the combined application of PRP and HA. Studies have shown that HA is not effective for patients with severe KOA disease, and the effect of HA decreases with time, especially in elderly patients [41, 42]. HA mainly provides nutrition and protection to joints and cannot regenerate damaged cartilage, while PRP contains a large number of growth factors that can promote chondrocyte proliferation and cartilage matrix synthesis [40]. Therefore, the combination of PRP and HA may have a better effect in patients who are elderly, have severe KOA, or exhibit a poor response to treatment with HA or PRP alone.

Nevertheless, several limitations were unavoidable. First, 2 articles were non-RCTs, which may have led to heterogeneity of the combined indicators. Second, the follow-up time was short, with the longest follow-up period being 1 year, and the long-term efficacy and safety of PRP combined with HA could not be evaluated. Third, from the indicators related to WOMAC, it was found that due to the inconsistent indicators reported in the literature, the combined results of the indicators related to WOMAC Pain Score and WOMAC Stiffness Score are lacking. In later clinical studies, attention should be paid to the comprehensiveness and consistency of the outcome indicators. Fourth, there are few studies that directly compare the efficacy and safety of the intra-articular injection of PRP combined with HA with those of the intra-articular injection of HA alone. Therefore, this meta-analysis failed to fully compare the efficacy of PRP combined with HA to that of HA alone. Fifth, the inclusion criteria and exclusion criteria of the included literature did not mention information about knee joint conditions, such as ligament instability, meniscus lesions, and alignment of the limb, which may affect the comparability of related data. Sixth, in terms of the KL Score evaluation, due to the limitation of the data included in the literature, the KL classification included grades I to IV, which may affect the credibility of the Lequesne Index evaluation.

## Conclusions

The results of this study indicate that PRP combined with HA may have promising clinical effects on KOA. Based on this meta-analysis, compared with intra-articular injection of PRP alone, PRP combined with HA can improve WOMAC Function Scores, WOMAC Total Scores, VAS ratings (after 6 months of treatment), and Lequesne Index scores. Additionally, in terms of the incidence of AEs, intra-articular injection of PRP combined with HA is not significantly different from intra-articular injection of PRP or HA alone, and the safety of the three treatment regimens is similar.

## Supplementary information

**Additional file 1.**

**Additional file 2.**

**Additional files 3.**

## Data Availability

All data and materials are contained within the manuscript.

## Abbreviations

PNAS: Proceedings of the National Academy of Sciences
PRP: Platelet-rich plasma
HA: Hyaluronic Acid
KOA: Knee Osteoarthritis
WOMAC: Western Ontario and McMaster Universities Arthritis Index
VAS: Visual analog scale
AEs: Adverse events
CNKI: China National Knowledge Infrastructure
RCT: randomized controlled trail
NOS: Newcastle-Ottawa Scale
RR: Relative risk
SMD: Standard Mean Difference
