# Supplementary material for: Effects and safety of the combination of platelet-rich plasma (PRP) and hyaluronic acid (HA) in the treatment of knee osteoarthritis: a systematic review and meta-analysis
Source: BMC Musculoskelet Disord. 2020 Apr 11;21:224. doi: 10.1186/s12891-020-03262-w (PMC7149899; doi:10.1186/s12891-020-03262-w)
Supplement: Supplementary file 1 — Additional file 1. [file 12891_2020_3262_MOESM1_ESM.docx]

Supplementary material 1

Pubmed

#1 ((("platelet-rich plasma"[Title/Abstract]) OR Plasma, Platelet-Rich[Title/Abstract]) OR "Platelet Rich Plasma"[Title/Abstract]) OR PRP[Title/Abstract]

#2 (((((((((((((("hyaluronic acid"[Title/Abstract]) OR HA[Title/Abstract]) OR Acid, Hyaluronic[Title/Abstract]) OR "Amo Vitrax"[Title/Abstract]) OR Vitrax, Amo[Title/Abstract]) OR Biolon[Title/Abstract]) OR Etamucine[Title/Abstract]) OR Hyaluronan[Title/Abstract]) OR Hyvisc[Title/Abstract]) OR Luronit[Title/Abstract]) OR "Sodium Hyaluronate"[Title/Abstract]) OR Hyaluronate, Sodium[Title/Abstract]) OR "Hyaluronate Sodium"[Title/Abstract]) OR Amvisc[Title/Abstract]) OR Healon[Title/Abstract]

#3 (((((("knee osteoarthritis"[Title/Abstract]) OR osteoarthritis, Knee[Title/Abstract]) OR "Knee osteoarthritides"[Title/Abstract]) OR "osteoarthritis of Knee"[Title/Abstract]) OR KOA[Title/Abstract]) OR "osteoarthritis of the Knee"[Title/Abstract]) OR osteoarthritis[Title/Abstract]

#4 #1 AND #2 AND #3

#4 (((((("platelet-rich plasma"[Title/Abstract]) OR Plasma, Platelet-Rich[Title/Abstract]) OR "Platelet Rich Plasma"[Title/Abstract]) OR PRP[Title/Abstract])) AND ((((((((((((((("hyaluronic acid"[Title/Abstract]) OR HA[Title/Abstract]) OR Acid, Hyaluronic[Title/Abstract]) OR "Amo Vitrax"[Title/Abstract]) OR Vitrax, Amo[Title/Abstract]) OR Biolon[Title/Abstract]) OR Etamucine[Title/Abstract]) OR Hyaluronan[Title/Abstract]) OR Hyvisc[Title/Abstract]) OR Luronit[Title/Abstract]) OR "Sodium Hyaluronate"[Title/Abstract]) OR Hyaluronate, Sodium[Title/Abstract]) OR "Hyaluronate Sodium"[Title/Abstract]) OR Amvisc[Title/Abstract]) OR Healon[Title/Abstract])) AND ((((((("knee osteoarthritis"[Title/Abstract]) OR osteoarthritis, Knee[Title/Abstract]) OR "Knee osteoarthritides"[Title/Abstract]) OR "osteoarthritis of Knee"[Title/Abstract]) OR KOA[Title/Abstract]) OR "osteoarthritis of the Knee"[Title/Abstract]) OR osteoarthritis[Title/Abstract])

Search results

Items: 1 to 20 of 170

Cochrane

#1(platelet-rich plasma):ti,ab,kw OR (Plasma, Platelet-Rich):ti,ab,kw OR (Platelet Rich Plasma):ti,ab,kw OR (PRP):ti,ab,kw

#2 ("hyaluronic acid"):ti,ab,kw OR (Acid, Hyaluronic):ti,ab,kw OR (HA):ti,ab,kw OR ("AMO Vitrax"):ti,ab,kw OR ("biolon"):ti,ab,kw

#3 (Etamucine):ti,ab,kw OR ("hyaluronan"):ti,ab,kw OR (HA):ti,ab,kw OR (Hyvisc):ti,ab,kw OR (Luronit):ti,ab,kw

#4 (Sodium Hyaluronate):ti,ab,kw OR (Hyaluronate, Sodium):ti,ab,kw OR (Hyaluronate Sodium):ti,ab,kw OR (Amvisc):ti,ab,kw OR (Healon):ti,ab,kw

#5 #2 OR #3 OR #4

#6 (knee osteoarthritis):ti,ab,kw OR ("knee osteoarthritides"):ti,ab,kw OR ("KOA"):ti,ab,kw OR ("osteoarthritis"):ti,ab,kw OR (osteoarthritis of Knee):ti,ab,kw

#7 #1 AND #5 AND #6

Embase date

#1 'platelet-rich plasma':ab,ti OR 'plasma, platelet-rich':ab,ti OR 'platelet rich plasma':ab,ti OR prp:ab,ti

#2 'hyaluronic acid':ab,ti OR 'acid, hyaluronic':ab,ti OR 'amo vitrax':ab,ti OR 'vitrax, amo':ab,ti OR biolon:ab,ti OR etamucine:ab,ti OR hyaluronan:ab,ti OR hyvisc:ab,ti OR luronit:ab,ti OR 'sodium hyaluronate':ab,ti OR 'hyaluronate, sodium':ab,ti OR 'hyaluronate sodium':ab,ti OR amvisc:ab,ti OR healon:ab,ti

#3 'knee osteoarthritis':ab,ti OR 'osteoarthritis, knee':ab,ti OR 'knee osteoarthritides':ab,ti OR 'osteoarthritis of knee':ab,ti OR 'osteoarthritis of the knee':ab,ti OR osteoarthritis:ab,ti OR koa:ab,ti

#4 #1 AND #2 AND #3

CNKI

（透明质酸[摘要] OR 玻尿酸[摘要]）AND 富血小板血浆[摘要] AND（膝关节炎[摘要] OR 膝骨关节炎[摘要]）
