# Supplementary material for: Effects and safety of the combination of platelet-rich plasma (PRP) and hyaluronic acid (HA) in the treatment of knee osteoarthritis: a systematic review and meta-analysis
Source: BMC Musculoskelet Disord. 2020 Apr 11;21:224. doi: 10.1186/s12891-020-03262-w (PMC7149899; doi:10.1186/s12891-020-03262-w)
Supplement: Supplementary file 2 — Additional file 2. [file 12891_2020_3262_MOESM2_ESM.docx]

Supplement 2 (PRP+HA)'s specific preparation process and medication details

| Author,year | (PRP+HA)'s specific preparation process | Injection Dose,Times,Intervals,Course of treatment | | | HA information |
| --- | --- | --- | --- | --- | --- |
|  |  | PRP+HA | PRP | HA |  |
| Yanqing Guo 2018 | Draw the prepared 3.5 ml PRP into a 10 ml syringe and connect it to a three-way connector. Connect the other end of the three-way connector to a 10 ml syringe containing 2 ml of HA, and mix the liquid by injecting it back and forth between the syringes 20 times. Yields 3.5 ml of PRP combined with 2 ml of HA (total volume =5.5 ml). | 3.5 ml of PRP combined with 2 ml of HA (total volume = 5.5 ml), 1 time per week, 3 weeks | 4-5ml,1 time,weekly,3 weeks | -- | natural,non-cross-  linked fermented HA (1550 kDa), concentration: 20 mg/ml (40 mg total) |
| Wenxing Yu 2018 | Combination (PRP: 8 ml,HA:0.20 mg).Not described in detail. | PRP:8 ml,HA:0.20 mg,1 time,weekly,4 weeks | 8 ml,1 time,weekly,4 weeks | 0.2 ml,1 time,weekly,4 weeks | 0.30 mg, Sigma-Aldrich; Merck  KGaA |
| Yanqing Guo 2018 | Draw the prepared 3.5 ml PRP into a 10 ml syringe and connect it to a three-way connector. Connect the other end of the three-way connector to a 10 ml syringe containing 2 ml of HA, and mix the liquid by injecting it back and forth between the syringes 20 times. Yields 3.5 ml of PRP combined with 2 ml of HA (total volume =5.5 ml). | 3.5 ml of PRP combined with 2 ml of HA (total volume = 5.5 ml), 1 time per week, 3 weeks | 4-5ml,1 time,weekly,3 weeks | -- | No detailed report. |
| Quanwei Ding 2017 | Mix activated PRP with HA in a disposable syringe.4 ml of PRP combined with 2.5 ml of HA(total volume =6.5 ml). | 4 ml of PRP combined with 2.5 ml of HA(total volume =6.5 ml),1 time,weekly,9 weeks | 4 ml,1 time,weekly,9 weeks | -- | 25 mg/2.5 ml ; Hyalgan, Fidia, Abano Terme, Italy |
| Xin-liang Zhao 2018 | After collecting 5 mL of the patient's own whole blood and centrifuging it to obtain PRP, mix the PRP with HA (2.5 mL:25 mg). Yields 4 ml of PRP combined with 2.5 ml of HA (total volume =6.5 ml). | 4 ml of PRP combined with 2.5 ml of HA (total volume =6.5 ml), 1 time per week, 5 weeks | 2 ml,1 time,weekly,5 weeks | -- | No detailed report. |
| Yanqing Guo 2018 | Inhale the prepared 3.5 ml PRP into a 10 ml syringe and connect it to a three-way connector. Connect the other end of the three-way connector to a 10 ml syringe containing 2 ml of hyaluronic acid, and repeatedly inject each other 20 times to mix.3.5 ml of PRP combined with 2 ml of HA(total volume =5.5 ml) | 3.5 ml of PRP combined with 2 ml of HA(total volume = 5.5 ml),1 time,weekly,3 weeks | 3.5 ml PRP,1 time,weekly,3 weeks | -- | No detailed report. |
| Chenrong Ke 2016 | PRP: Use a 50 ml syringe preloaded with 4 ml sodium citrate anticoagulant to draw 40 ml of blood from the cubital vein. Shake the sample, place it in a special centrifuge tube, and centrifuge at 1,500 r/min for 10 min (centrifugation radius 19 cm). Use a pipette to transfer the supernatant and the 3 mm liquid (serum and PRP) just below the interface to another sterile centrifuge tube containing no anticoagulant, and then centrifuge it again at 1,500 r/min for 10 min. Remove the upper 3/4 of the liquid after centrifugation; the remaining liquid is PRP. Activate and prepare 4 ml PRP by adding 0.2 ml calcium chloride.  Combined injection method: First, inject 4 ml of prepared PRP into the joint cavity; 10 min later, inject 2 ml of HA. After the injection is completed, slowly flex and extend the knee joint. Uses 4 ml of PRP combined with 2 ml of HA (total volume =6 ml). | 4 ml of PRP combined with 2 ml of HA (total volume =6 ml), 1 time per week, 5 weeks | 6 ml PRP,1 time,weekly,5 weeks | 6 ml HA,1 time,weekly,5 weeks | No detailed report. |
