# Supplementary material for: Effects and safety of the combination of platelet-rich plasma (PRP) and hyaluronic acid (HA) in the treatment of knee osteoarthritis: a systematic review and meta-analysis
Source: BMC Musculoskelet Disord. 2020 Apr 11;21:224. doi: 10.1186/s12891-020-03262-w (PMC7149899; doi:10.1186/s12891-020-03262-w)
Supplement: Supplementary file 3 — Additional files 3. [file 12891_2020_3262_MOESM3_ESM.docx]

Supplement 3

1.VAS after 1 month of treatment

Tests for Publication Bias

Begg's Test

adj. Kendall's Score (P-Q) = -1

Std. Dev. of Score = 1.91

Number of Studies =3

z =-0.52

Pr > |z| =0.602

z = 0.00 (continuity corrected)

Pr > |z| =1.000 (continuity corrected)

Egger's test

Std_Eff | Coef. Std. Err. t P>|t| [95% Conf. Interval]

slope | 1.076847 9.540671 0.11 0.928 -120.1489 122.3026

bias | -8.584955 37.64227 -0.23 0.857 -486.8754 469.7055

2.VAS after 6 months of treatment

Tests for Publication Bias

Begg's Test

adj. Kendall's Score (P-Q) =-2

Std. Dev. of Score =2.94

Number of Studies =4

z=-0.68

Pr > |z| =0.497

z =0.34 (continuity corrected)

Pr > |z| = 0.734 (continuity corrected)

Egger's test

Std_Eff | Coef. Std. Err. t P>|t| [95% Conf. Interval]

slope | -.1285936 .2060591 -0.62 0.596 -1.015194 .7580069

bias | -.5816812 .9974241 -0.58 0.619 -4.873251 3.709888

3.Adverse events (PRP+HA VS PRP)

Tests for Publication Bias

Begg's Test

adj. Kendall's Score (P-Q) = -6

Std. Dev. of Score = 4.08

Number of Studies = 5

z = -1.47

Pr > |z| =0.142

z= 1.22 (continuity corrected)

Pr > |z| = 0.221 (continuity corrected)

Egger's test

Std_Eff | Coef. Std. Err. t P>|t| [95% Conf. Interval]

slope | .7890938 .5871655 1.34 0.272 -1.079529 2.657716

bias | -1.210074 .8944884 -1.35 0.269 -4.056735 1.636588
